# An economical and feasible method with little trauma for a giant pancreatic pseudocyst communicating with main pancreatic duct: a case report and literature review

**DOI:** 10.3389/fmed.2025.1666692

**Published:** 2025-12-16

**Authors:** Minjing Luo, Yuxiang Kuang, Shijuan Luo, Yiqun Lin, Zishao Zhong, Zhenhao Ye

**Affiliations:** 1 Department of Gastroenterology, The Second Affiliated Hospital of Guangzhou University of Chinese Medicine, Guangzhou, China; 2 Department of Gastroenterology, Guangdong Provincial Hospital of Chinese Medicine, Guangzhou, China

**Keywords:** giant pancreatic pseudocyst, economical and feasible method, transpapillary drainage, a case report, literature review

## Abstract

Pancreatic fluid collection is a common complication of both acute and chronic pancreatitis, often presenting as pancreatic pseudocysts (PPCs) or walled-off pancreatic necrosis. Treatment options for PPCs include percutaneous catheter drainage (PCD), surgical drainage (SD), and endoscopic drainage (ED). However, due to the lack of large studies and prospective randomized data, the optimal approach for managing large PPCs remains controversial. Here, we report a case of a patient with a documented history of acute pancreatitis who developed a PPC measuring approximately 5.9 cm × 4.9 cm × 7.7 cm, located posterior to the pancreatic head. He underwent a combined endoscopic and nutritional strategy that involved pancreatic duct stenting for internal drainage, along with the placement of a nasojejunal tube for enteral feeding. After 2 months, the patient showed clinical improvement and a reduction in the size of his PPC on imaging studies. This case highlights that the implantation of a pancreatic duct stent, combined with a nasojejunal nutrition tube, may offer a cost-effective and minimally invasive approach for managing large PPCs that communicate with the main pancreatic duct.

## Introduction

Pancreatic fluid collection is a common complication of both acute and chronic pancreatitis. It presents as pancreatic pseudocysts (PPCs) and walled-off pancreatic necrosis, a type of pancreatic cystic disease (1). Since PPCs typically arise following pancreatitis, their causes and epidemiological patterns are quite similar. The incidence of PPCs is higher in men, with alcohol consumption implicated in over 70% of cases (2). PPCs are more likely to develop from chronic pancreatitis, with incidence rates ranging from 20–40%, compared to 5–16% for acute pancreatitis. This difference is likely due to the increased likelihood of duct damage associated with prolonged pancreatic inflammation (3). Overall, regardless of the underlying cause, the incidence of PPCs is estimated at 0.5–1 per 100,000 people per year (4).

Improper handling of PPCs can lead to serious complications, including infected pancreatic necrosis (IPN), intracystic, intraperitoneal, or gastrointestinal bleeding, as well as pancreatic and intestinal fistulas. Treatment options for PPCs include percutaneous catheter drainage, laparoscopic surgical intervention, and Roux-en-Y anastomosis of the cyst to the jejunum (5, 6). Endoscopic retrograde cholangiopancreatography (ERCP) drainage is also utilized. Current guidelines emphasize strong evidence and recommendations for endoscopic ultrasound-guided puncture drainage, particularly recommending the use of lumen-apposing metal stents (LAMS) (7). However, the placement of these stents carries risks, such as bleeding, displacement, detachment, and embedding (8).

In this study, we present a case of a giant PPC communicating with the main pancreatic duct, identified through imaging examination, and successfully managed by pancreatic duct stent implantation combined with a naso-jejunal nutrition tube.

## Case report

**Figure 1:**
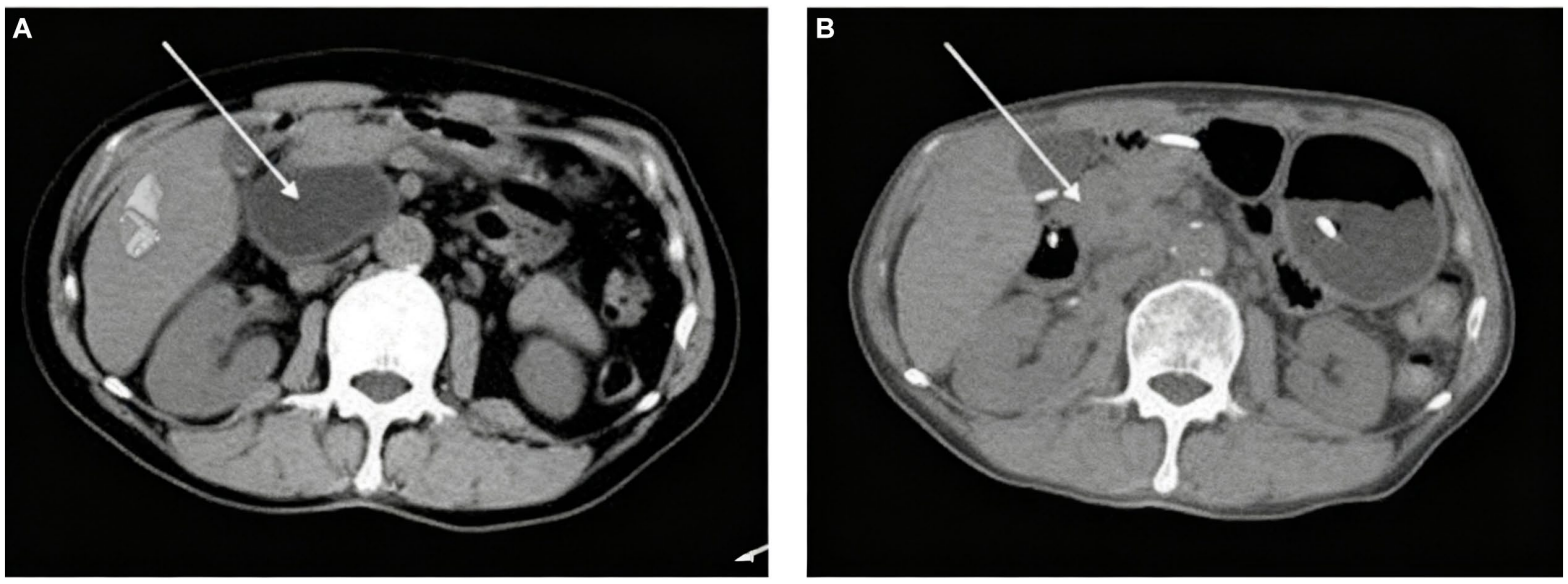
Abdominal CT scans demonstrate the **(A)** pancreatic enlargement with a cystic hypodence lesion posterior to the pancreatic head; **(B)** reduction in the size of pancreatic cystic lesion after 2 months.

**Figure 2:**
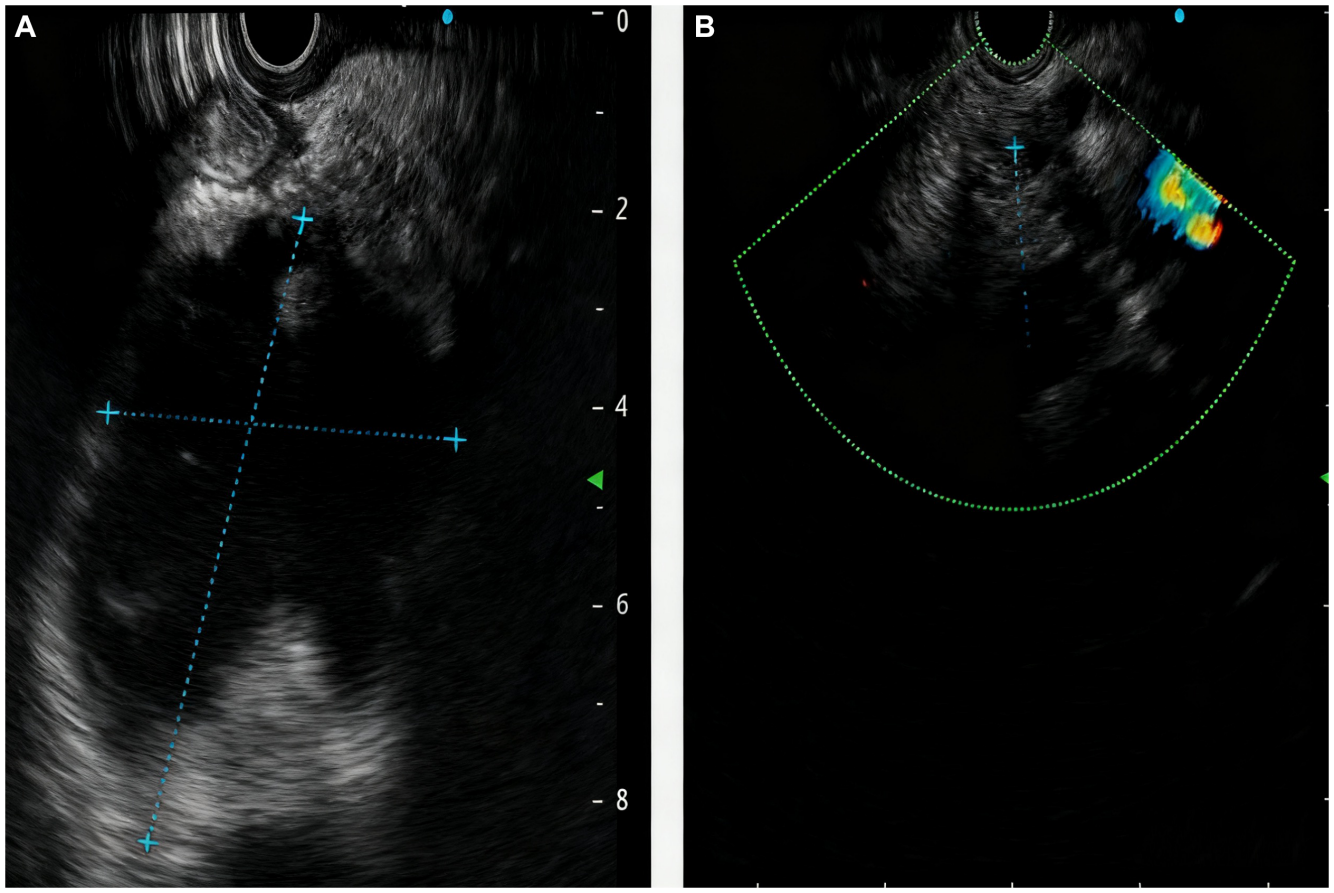
The manifestations of the pancreas under endoscopic ultrasound revealed the **(A)** a cystic lesion measured approximately 6.7cm x 5.4cm in its largest cross-sectional dimension, communicated with the MPD; **(B)** reduction in the size of pancreatic cystic lesion after 2 months.

A 64-year-old male of Asian with a documented history of acute pancreatitis 11 months prior presented with recurrent severe epigastric pain, a CT scan that revealed pancreatic enlargement with ill-defined margins and heterogeneous density, along with a cystic hypodense lesion measuring approximately 5.9 cm × 4.9 cm × 7.7 cm posterior to the pancreatic head. This lesion showed slightly thickened walls without mural nodules, had an indistinct border with the pancreatic head, and was accompanied by pancreatic duct dilation at 9 mm and ill-defined fat planes surrounding both the pancreas and duodenum (Figure 1A). The endoscopic ultrasonography demonstrated that a cystic lesion was identified in the head and neck of the pancreas measured approximately 6.7 cm × 5.4 cm in its largest cross-sectional dimension, without definite mural nodule. The main pancreatic duct (MPD) distal to the cyst is dilated, measuring up to 0.7 cm in diameter, and communicated with the cyst (Figure 2A). Upon initial evaluation, he was noted to be underweight with a body-mass index (BMI) of 16.29 kg/m^2^. Laboratory results showed increased amylase of 175 U/L, lipase of 76 U/L, and alpha-fetoprotein (AFP) of 14.3 ng/mL. Carcinoembryonic antigen (CEA), CA 19–9, total bilirubin, alanine aminotransferase (ALT) and aspartate aminotransferase (AST) were all within normal limits.

Given his unfavorable performance status and his poor nutritional status, as well as the specific manifestation of a cyst communicating with the MPD, the patient was not considered to be a candidate for percutaneous drainage or surgical necrosectomy in such large pancreatic pseudocyst. Rather, he received a combined endoscopic and nutritional strategy involving pancreatic duct stenting for internal drainage alongside nasojejunal tube placement for enteral feeding.

**Figure 3:**
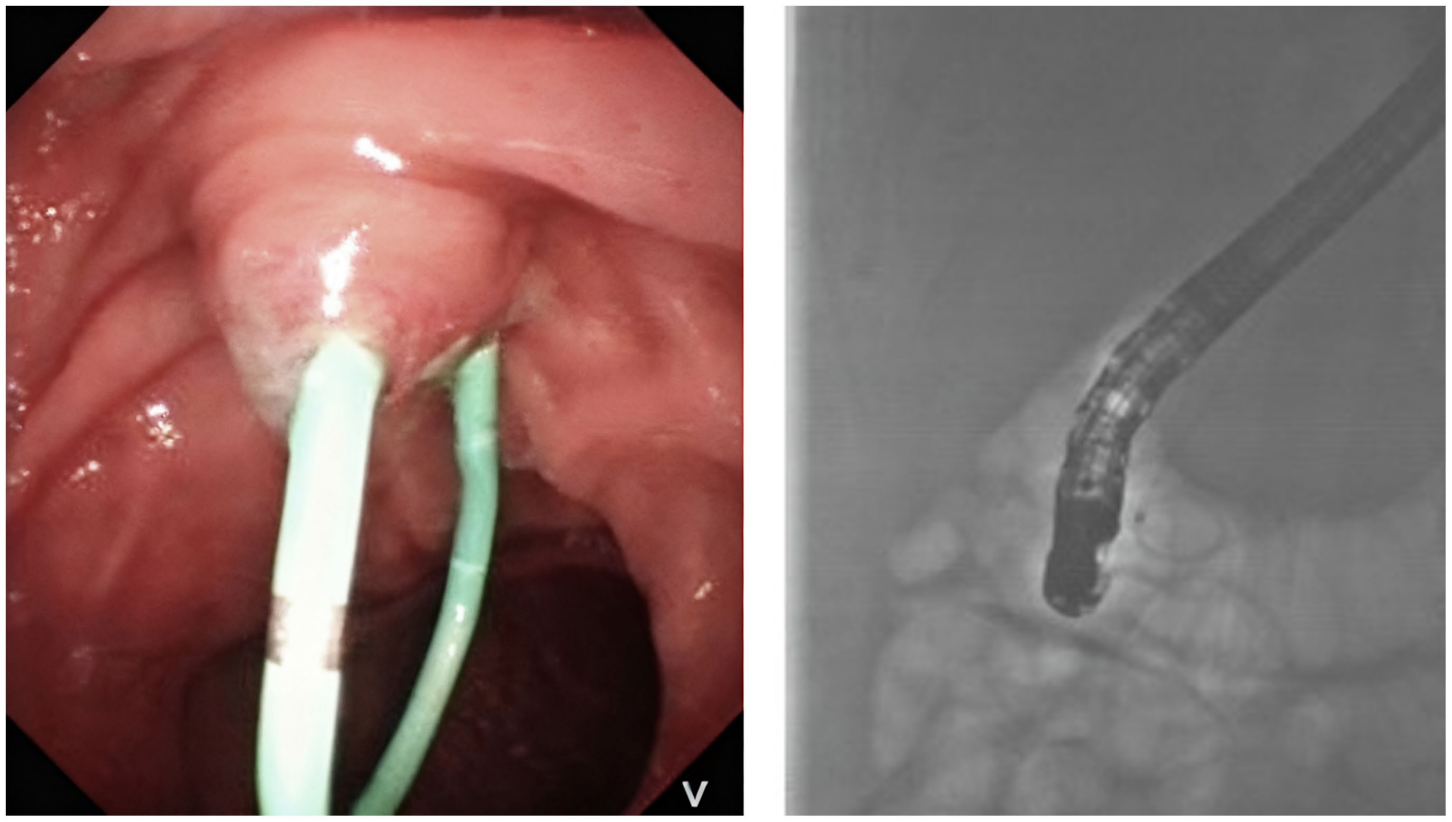
The process of inserting a papillary pancreatic duct stent.

We utilized a duodenoscope to access the descending part of the duodenum, where we observed a single-hole type duodenal papilla. A three-chambered knife was employed to carry a guidewire and insert the tube, successfully entering the pancreatic duct. We maintained the guidewire in the pancreatic duct while injecting contrast agent through the three-chambered knife. This allowed us to visualize the main pancreatic duct, which measured approximately 1 cm in diameter. After the contrast injection, we aspirated the contrast agent from the pancreatic duct and placed a 5 Fr × 5 cm plastic pancreatic duct stent (Boston Scientific Jagwire™ SPSOF-5-5) in the duct. Upon stent insertion, a significant amount of white flocculent material was noted to flow out. The bile duct was not visualized during the procedure (Figure 3). Additionally, we inserted a nasojejunal nutrition tube. The patient’s vital signs remained stable upon completion of the procedure. This dual approach facilitated effective cyst decompression while simultaneously attenuating pancreatic auto-digestion by reducing secretory stimulation.

**Figure 4:**
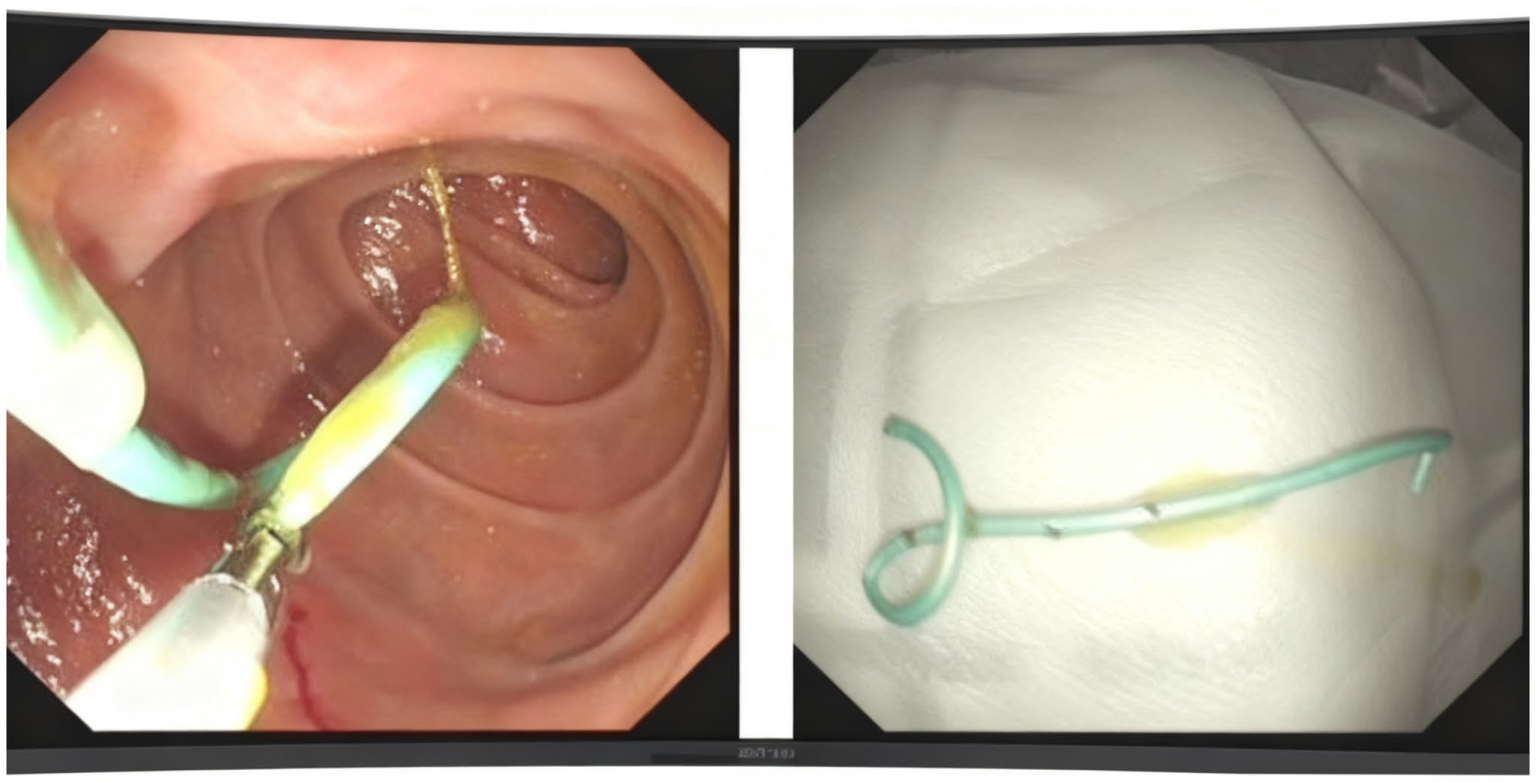
The process of moving the stent.

After two-month follow-up, we removed the pancreatic duct stent (Figure 4). His BMI had improved at 17.56 kg/m^2^, findings compatible with clinical improvement. Imaging studies both in CT scan and endoscopic ultrasonography confirmed reduction in the size of his PPC (measuring 2.2 cm × 0.8 cm × 1.1 cm) and his previously mentioned elevated laboratory values were all within normal limits (Figures 1B, 2B).

## Discussion

A PPC is typically diagnosed using ultrasound, CT scans, or magnetic resonance imaging (MRI). High-resolution endoscopic ultrasound (EUS) is particularly effective at detecting cystic lesions smaller than 2 cm in diameter, offering high diagnostic sensitivity. In addition, ERCP is a crucial tool not only for diagnosing pseudocysts but also for facilitating endoscopic treatment (9). The management of all PPCs is influenced by their size and morphology. Expert consensus guidelines have been established for managing pancreatic cysts, identifying specific morphologic features to classify cysts into two categories: those with “worrisome features” and those without. Worrisome features include: (1) cyst size large than 3 cm, (2) thickened or enhancing cyst walls, (3) non-enhancing mural nodules, and (4) main pancreatic duct caliber >5–9 mm (10). Careful imaging evaluation of incidental pancreatic cysts is essential, as the cyst’s characteristics play a critical role in determining management strategies. For instance, surveillance is generally advised for cysts smaller than 3 cm without worrisome features or high-risk indicators (11, 12). Conversely, cysts exhibiting worrisome features should undergo sampling via endoscopic ultrasound fine-needle aspiration (EUS-FNA) (13, 14), while those with high-risk stigmata are usually recommended for surgical resection.

PPC treatment options, which include percutaneous catheter drainage (PCD), surgical drainage (SD), and endoscopic drainage (ED), currently favor ED as the optimal approach. ED can be categorized into two types: transmural drainage (TMD) and transpapillary drainage (TPD). The choice between these methods depends on the location of the cyst, its connection to the MPD, any ductal obstruction present, and the physician’s expertise. TPD involves balloon dilation and stenting performed under endoscopic retrograde pancreatography (ERP) and requires that there be communication between the MPD and the PPCs. In contrast, TMD can be performed across the duodenal or gastric wall under EUS, necessitating that the PPCs are located close to the gastrointestinal wall (within 1 cm). Studies by Trevino et al. and Shrode et al. had shown that TPD can improve treatment outcomes for TMD in cases with partial disruptions of the pancreatic duct associated with pancreatic fluid collections (PFCs) (15, 16). Additionally, recent research by Ni et al. suggested that transpapillary pancreatic duct stenting enhances the efficacy of endoscopic TMD for PFCs related to pancreatic duct disruptions, resulting in a lower recurrence rate and a shorter hospital stay (17). Moreover, Libera et al. found that various endoscopic drainage methods, including TMD, TPD, and catheter drainage (CD), do not significantly differ in their effectiveness for treating PPCs (18). However, Hookey et al. highlighted that using both transpapillary and transmural techniques together may lead to a higher recurrence rate compared to performing only transmural drainage (19). Currently, we have reviewed ten reports on the treatment of large PPCs, and the benefits and risks of these options remain controversial, as outlined in Table 1. There is general consensus among clinical experts that patients displaying certain characteristics are more likely to qualify for surgical intervention. These characteristics include pseudocysts that cause duodenal obstruction, biliary compression, pancreatic ascites, or pancreatic fistula, as well as infected pseudocysts, hemorrhage, severe symptoms, and asymptomatic pseudocysts larger than 6 cm. However, surgical management tends to be associated with longer hospital stays and a higher risk of significant complications.

**Table 1:** Overview of case reports for treatment on large pancreatic pseudocysts.

Report	Imaging examination manifestations	Treatment	Complications	Results
Groskreutz et al. (20)	A 10.2 × 10 cm multiloculated fluid collection extending from the head of the pancreas to the lower right psoas muscle, deviating the right colon.	Interventional radiology placed a 12 F drain for draining fluid.	Secondary infection.	Recovery
Nalwa et al. (21)	A sizeable septated pancreatic cyst with measurements of 15.8 cm × 14 cm × 14 cm present near the tail of the pancreas with no mural nodules and calcifications.	Percutaneous drainage of the pancreatic pseudocyst.	No significant reduction in the mass with septated cystic structure.	Referral to cystogastrostomy and discharge from hospital.
Billari et al. (22)	Atrophy of the pancreas with calcifications, large developing fluid collection in the left upper quadrant of the abdomen estimated up to 22 cm maximal dimension with regional mass effect.	Endoscopic ultrasound-guided cystogastrostomy with later removal of the stent and placement of the pigtail catheter for continued drainage.	Prolonged hospital stay.	Recovery
Supapannachart et al. (23)	A pancreatic cyst measuring 15.9 × 10.4 cm displaced the stomach and compressed the splenic vasculature.	Endoscopic cystogastrostomy stent placement.	Secondary infection and acute hypoxemic respiratory failure with a large left pleural effusion.	Open laparotomy and discharged after protracted postoperative course.
Chen et al. (24)	Pancreatic pseudocysts measurements of 4.7 cm × 3.8 cm with the cystic heterogeneous hypoechoic area located posterior to the body and tail of pancreas, and adjacent to splenic vein associated with thrombosis resulted from compression.	Distal pancreatectomy combined with splenectomy and partial gastrectomy.	NA	Manifested stable condition.
Udeshika et al. (25)	A large multilocular cystic lesion measured 30 cm × 15 cm × 14 cm displacing the liver medially and the right dome of the diaphragm superiorly with two smaller locules, contained clear fluid with no enhancement of the walls.	Endoscopic ultrasound guided drainage of the cyst.	NA	Recovery
Alhassan et al. (26)	A pancreatic pseudocyst measurement of 20 × 12 × 10 cm.	Percutaneous CT-guided cyst drainage.	Prolonged hospital course with multi-organ failure, mechanical ventilation use, total parenteral nutrition.	Laparotomy with pancreatic necrosectomy and discharge from hospital.
Wei et al. (27)	Multiple sizable PPCs throughout the pancreas, including a large one (122 × 79 mm) communicating with the tail.	EUS-TMD with a lumen-apposing metal stent.	The ENPD drainage duct obstructed lead to secondary infection.	Discharged from the hospital with stabilized condition.
Gu and Qian (28)	Multiple small cystic lesions in the head of the pancreas, the biggest on measured 1.2 × 1.1 cm, and a 6.5 × 5.7 cm cystic lesion adherent to the tail of the pancreas.	Bile duct stent and pancreatic duct stent placement was performed endoscopicly.	NA	Recovery
Krajewski et al. (29)	A 100-mm pseudocyst in the tail of pancreas.	Endoscopic transmural drainage.	Profuse submucosal bleeding at the puncture site.	Distal pancreatectomy with splenectomy and discharge from hospital.

NA, not applicable; CT, computerized tomography; EUS-TMD; endoscopic ultrasound-guided transmural drainage.

We would like to emphasize the relevance and therapeutic implications of the case presented. Unlike other reports on the treatment of large PPCs, this patient’s poor general condition improved significantly with a combined approach involving the implantation of a pancreatic duct stent and the placement of a naso-jejunal nutrition tube. This clinical improvement, evidenced by the recovery of the patient’s performance and nutritional status, was attributed to the protective effects of the naso-jejunal nutrition tube and a positive drainage response. TPD is a viable option for managing PPCs resulting from chronic pancreatitis, as the MPD often connects with the cyst. By using a pancreatic duct stent, a pathway can be created between the cyst and the duodenal lumen. The currently used stents for endoscopic drainage include plastic stents and full-coated self-expanding metal stents that adhere to the cavity wall (LAMS). The double-tailed plastic stent was the earliest used for transmural drainage in PPC, and its efficacy for PPC drainage exceeded 90%. The LAMS release device can be guided by a guidewire and burn the tip step-by-step to the cystic cavity. It is released under the monitoring of EUS and X-ray, allowing the cyst wall and the gastrointestinal wall to closely adhere to each other, thereby reducing the occurrence of stent displacement. Current studies have shown that there are no significant differences in treatment success rate, re-intervention rate, adverse events, and hospital stay between LAMS and plastic stents for treating PPC, but the price is five times that of the plastic stent. Because of poor economic status of our patient, an ERCP was performed with plastic pancreatic duct stent, which revealed clear and transparent pancreatic juice without any mucus and blood, thereby ruling out the possibility of an intraductal papillary mucinous neoplasm (IPMN).

The large size and trans-spatial characteristics of the PPC, combined with relatively benign symptoms and eventual shrinkage, highlight the unique aspects of this case. The use of pancreatic duct stent implantation, along with a naso-jejunal nutrition tube, may represent a cost-effective and minimally invasive approach for managing a large PPC that communicates with the MPD.

## Data Availability

The datasets presented in this study can be found in online repositories. The names of the repository/repositories and accession number(s) can be found in the article/supplementary material.
